# Hydatid cyst involving Right Pectoralis Major Muscle: A case report

**DOI:** 10.1016/j.ijscr.2019.04.004

**Published:** 2019-04-06

**Authors:** ZakaUllah Jan, Salma Zeb, Azam Shoaib, Kaleem Ullah, Muhammad Muslim, Humaira Anjum, Hina Wazir, Mahmud Aurangzeb

**Affiliations:** a Department of General Surgery, Khyber Teaching Hospital Peshawar, Pakistan; b Department of Radiology, Khyber Teaching Hospital Peshawar, Pakistan; c Geisinger Commonwealth School of Medicine 525 Pine St, Scranton, PA 18510, USA

**Keywords:** Hydatid cyst, Pectoralis major, Echinococcosis

## Abstract

**Background and aim:**

Hydatid disease is caused by Echinococcus granulosus; a tapeworm. They rarely affect the musculoskeletal system of the body (<5% of cases). The objective of this case report is to discuss about hydatid cyst disease involving the right pectoralis major muscle.

**Case presentation:**

Herein, we report a case of a 35 years old lady who came to the outpatient department with a gradually increasing soft swelling at the right upper chest below the clavicle. The final diagnosis was made with the help of a CT scan. The cyst was excised and the area was washed with a scolicidal agent. There was no recurrence after six months of follow up and the patient was doing well.

**Conclusion:**

Hydatid cyst of the Pectoralis Major is rare and must be considered in the differential of any muscular swelling in the body in endemic areas. These cysts should be treated with enucleation/excicion followed by irrigation of the area with a scolicidal agent.

## Highlights

• Hydatid cyst is fairly common in endemic areas & can involve any part of the body.
• Involvement of Pectoralis major muscle is rare.
• Ultrasound and CT scan help in diagnosing them.
• Most of the times, surgery is the usual treatment modality, followed by irrigation with a scolicidal agent.
• A course of albendazole should be prescribed after surgery.
• Hydatid cyst is fairly common in endemic areas & can involve any part of the body.
• Involvement of Pectoralis major muscle is rare.
• Ultrasound and CT scan help in diagnosing them.
• Most of the times, surgery is the usual treatment modality, followed by irrigation with a scolicidal agent.
• A course of albendazole should be prescribed after surgery.

## 1 Introduction

Hydatid disease is a major problem of public health in areas that are endemic including Asia, Africa, the Middle East, Mediterranean countries, Australia and South America. [1] Echinococcosis is caused by a tapeworm belonging to the cestode class called Echinococcus and liver and lungs are the commonly affected organs of the body in 75% and 15% of cases respectively. [2,3] Due to filtering action of liver and lungs in preventing echinococcus to enter systemic circulation, [4] contractility of muscles and high concentration of lactic acid [5,6], hydatid cyst is rarely found in the muscles and the reported incidence is less than 5% [7].

We present a case of a middle aged lady with a hydatid cyst of pectoralis major muscle. This is rarely mentioned in the literature. The aim of this study is to highlight this rare presentation and discuss the treatment modalities.

This work has been reported in line with the SCARE criteria. [8]

## 2 Case presentation

**Figs. 1–3:**
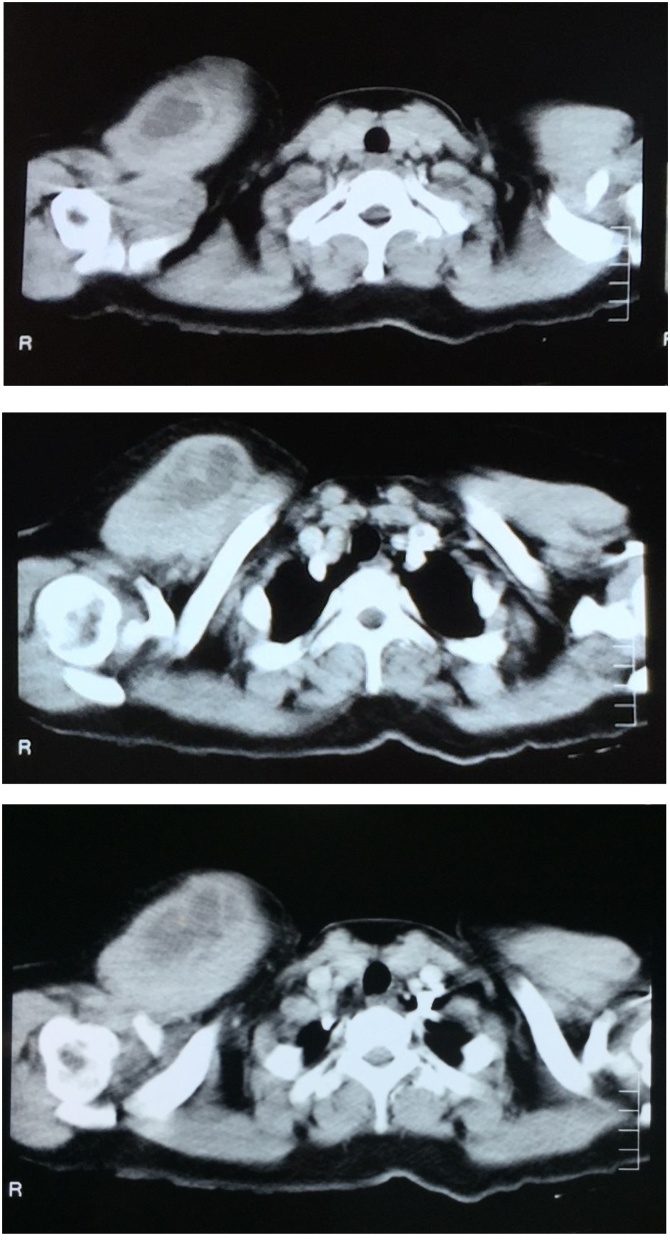
Showing Hydatid cyst in Right Pectoralis major muscle.

A female patient, 35 years of age, presented to the outpatient department with the chief complaints of a painless cystic swelling over the right lateral upper chest area 3 cm below the clavicle for the last 2 and a half years. According to the patient, the swelling was initially small but gradually increased in size over the mentioned course of period. The patient denied any history of pain, fever or any trauma. Her family, drug, or psychosocial history including smoking status was unremarkable. On examination, there was a 6 x 8 cm soft non-tender cystic swelling at the right upper chest region. The overlying skin was intact. Initial investigations were normal. On further work up, a computed tomography scan of the chest demonstrated a multiseptated hypodense cystic lesion in the right pectoralis major muscle with no intralesional calcification and no intrathoracic extension (Figs. 1–3).

**Fig. 4:**
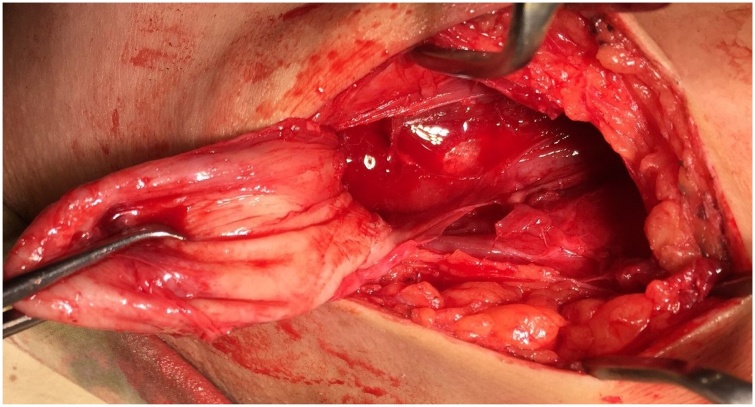
Showing Hydatid cyst attached to the right pectoralis major muscle.

**Fig. 5:**
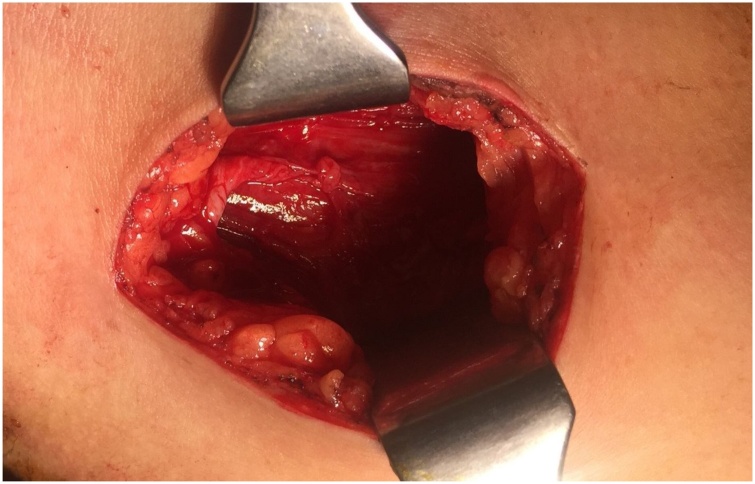
Showing Right Pectoralis Major muscle free of hydatid cyst.

**Fig. 6:**
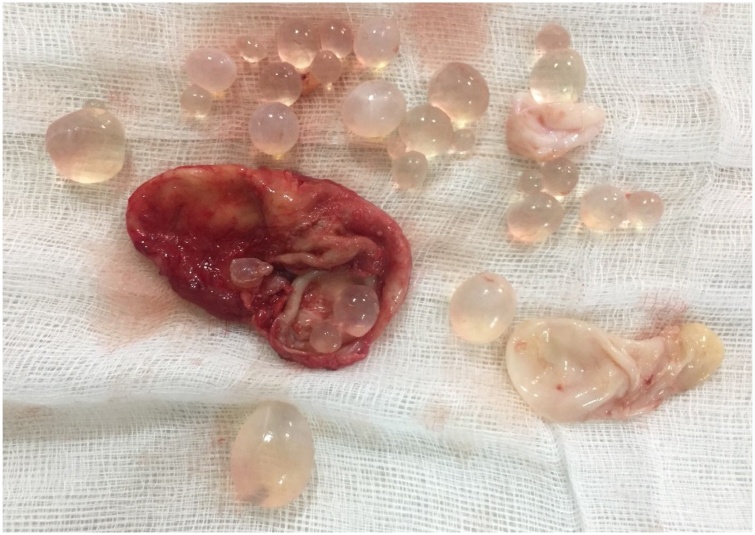
Showing excised hydatid cyst along with beautiful grapes like daughter cysts.

Surgery was planned and was performed by the consultant. Per-op finding revealed a 6 x 7 cm hydatid cyst in the right pectoralis major muscle with multiple daughter cysts (Fig. 4, Fig. 5, Fig. 6).

Under general anesthesia, she underwent excision of the cyst that was followed by wash of the cavity with hypertonic saline. A suction drain was placed and removed after a period of 24 hrs. The patient made an uneventful recovery post-operatively and stayed in the unit for a day. She was put on oral course of tablet albendazole and was sent home the next day. On 6 months follow up, the patient was doing well and had no complaints or any recurrence.

## 3 Discussion

Hydatidosis is a health problem in developing countries. This is due to a number of reasons such as illegal slaughtering of animals, high no of stray dogs and lack of proper public awareness regarding hydatid disease. [9] Infection occurs when the ova are ingested by the definitive host (dog). In the intermediate host (man), these ova hatch into embryos in the duodenum as they are resistant to hydrochloric acid in the stomach. These embryos penetrate the mucosa of the duodenum into a portal vein tributary and thus transported into liver [10]. Hydatid cysts most commonly occur in liver and the lungs. Musculoskeletal hydatidosis is rare. To the best of the author’s knowledge, hydatid cyst of the pectoralis major muscle was first reported by Abdel-Khaliq [10]. It has also been reported in various other anatomical locations encompassing biceps femoris, triceps, supraspinatus muscles, diaphragm, psoas, sartorius, thigh, quadriceps femoris and gracilis [11]. Mseddi et al. [12] over a period of 17 years reported 11 hydatid cysts in proximal muscles of the limbs. Khanna et al. [13] conducted a study of 110 patients over a period of 23 years and reported 24 various unusual locations of hydatid cysts including spleen followed by skin and soft tissue.

Initially, hydatid cyst is asymptomatic. But as the cyst grow in size, patient presents with various signs and symptoms depending on site of localization, the organ involved, its effect on the nearby structures, secondary infection, immunological reactions and complications secondary to rupture. Clinically, a hydatid cyst involving a muscle presents as a palpable mass and the symptoms are due to compression of the involved organ. [[14], [15], [16]] Our patient presented with a gradually increasing mildly tender swelling in right pectoralis major muscle.

Imaging techniques play a vital role in diagnosing echinococcosis and are used to detect these cystic lesions. [17] Ultrasound and CT scan are best for diagnosis as reported by Dancie et al. [18] and Moumen et al. [19] Ultrasound is cheap, non-invasive and can be repeated if necessary. On ultrasound; detached membranes, daughter cysts and double-line sign are the characteristics of a hydatid cyst [20]. On computed tomography scan, hydatid cyst can appear variably as an atypical complex, as a unilocular cyst, or a multivesicular lesion; that being the distinguishing feature which shows multiple daughter cysts inside the parent cyst. As with ultrasound, the appearance of detached membranes and daughter cysts on CT scan may help in the diagnosis. Moreover, computed tomography scan has an advantage in demonstrating bony associations and wall calcifications [21,22].

The treatment of hydatid cyst is either medical or surgical. The surgical approach should be tailored according to the cyst features. However, in toto removal of the cyst is preferred, if possible. Surgery encompasses either enucleation or resection of the cyst and washing the cavity with a scolicidal agent e.g; hypertonic saline, hydrogen peroxide, formaldehyde, ether,

alcohol and cetrimide for prevention of recurrence [22,23]. In our case, the patient underwent first excision of the cyst and then sterilization of the residual cavity with hypertonic saline.

## 4 Conclusion

Echinococcosis can affect any organ of the body. As discussed earlier, hydatid cyst involving the pectoralis major muscle is rare. Therefore, hydatid cyst must be taken into consideration in the differential for a patient who presents with a cystic swelling involving the musculoskeletal system of the body especially in endemic areas. Imaging is helpful in the diagnosis. The treatment is surgical followed by sterilization of the cavity with a scolicidal agent.

## Disclosure

This case was presented as a video presentation in the “International Conference on Surgery and Anesthesia” held during August 06-07, 2018 in Tokyo, Japan.

## Conflicts of interest

There are no conflicts of interests.

## Funding

There are no sources of funding. The authors are paying for the publication fee.

## Ethical approval

Khyber Teaching Hospital Peshawar, ethical committee.

## Consent

A written informed consent was obtained from the patient for publication of this case report and accompanying images.

## Author contribution

ZakaUllah Jan: Wrote the paper and assisted the consultant in the procedure.

Azam Shoaib: Prepared the patient pre-operatively and assisted the consultant in the procedure.

KaleemUllah: Participated in data collection.

Muhammad Muslim: Performed the procedure.

Humaira Anjum: Reported the CT scan.

Hina Wazir: Data analysis and interpretation.

Salma Zeb: Study concept and data collection.

Mahmud Aurangzeb: Supervised the whole team.

## Registration of research studies

NA.

## Guarantor

ZakaUllah Jan.

## Provenance and peer review

Not commissioned, externally peer-reviewed
